# Short-term outcomes following total correction of tetralogy of fallot in adult patients

**DOI:** 10.1186/s13019-023-02411-1

**Published:** 2023-11-14

**Authors:** Zahra Khajali, Nasibeh Mohammadi, Yaser Toloueitabar, Majid Maleki, Sedigheh Saedi, Zeinab Norouzi, Saeedeh Mazloum-Zadeh, Maryam Chenaghlou, Amirhossein Jalali, Hassan Tatari, Maryam Aliramezany

**Affiliations:** 1 Rajaei Cardiovascular, Medical and Research Center, Iran University of Medical Sciences, Tehran, Iran; 2 Department of Cardiology, Zanjan University of Medical Sciences, Zanjan, Iran; 3 Cardiovascular Research Center, Tabriz University of Medical Sciences, Tabriz, Iran; 4 Cardiovascular Research Center, Institute of Basic and Clinical Physiology Sciences, Kerman University of Medical Sciences, Kerman, Iran

**Keywords:** Tetralogy of Fallot, Tetralogy of Fallot Total correction, Congenital Heart Disease, Adulthood, Mortality, Outcome, Complication

## Abstract

**Objectives:**

Tetralogy of Fallot (TOF) is a common congenital heart disease which should be corrected. The recommended time for the Tetralogy of Fallot Total Correction (TFTC) surgery is during the infancy for the possible difficulties during the surgery and the related issues. However, sometimes TOF is diagnosed and managed during the adulthood.

**Methods:**

This study is a descriptive and retrospective one which included all patients who underwent TFTC at the age of 15-year and older in 10 years (between the years 2010 and 2020) to identify short-term (in-hospital mortality, ICU stay, postoperative bleeding, respiratory complications after the surgery such as pulmonary edema, pneumonia, etc.) and one-year (left ventricle ejection fraction (LVEF), right ventricle (RV) ejection fraction, the severity of tricuspid and aortic regurgitation after surgery) outcomes. All data were taken from medical records at Rajaie Cardiovascular Medical and Research Center. Data were analyzed using SPSS 22.

**Results:**

94 patients with the mean ± SD age of 26.7 ± 9.6 years were enrolled. Most of them were male (59.6%) (P-value: 0.009). In-hospital mortality in our study were 5.3%. Tricuspid regurgitation (TR) was significantly resolved after the surgery (P-value: 0.006). Of 17 (18.1%) patients with small or hypoplastic pulmonary artery (PA) branches, 14 patients had acceptable PA branch size after surgery.

**Conclusion:**

TFTC at an older age is safe with acceptable results. Age is not a contraindication for TFTC and surgery should be recommended if the patients are diagnosed with TOF in adulthood. Also, the TOF diagnosis should be considered in adult patients with suspicious signs and symptoms.

## Introduction

Tetralogy of Fallot (TOF) a common congenital heart disease (CHD) [1], accounts for 7–10% of all congenital heart anomalies and can be cyanotic or acyanotic [2]. This disease includes four underlying structural abnormalities including pulmonary artery stenosis (PS), ventricular septal defect (VSD), right ventricular hypertrophy (RVH), and overriding of the aorta which is the result of anterior and superior deviation of infundibular septum [3].

In developed countries, TOF is usually diagnosed and treated at early ages by a surgery called Tetralogy of Fallot Total Correction (TFTC). In those settings, primary repair is the strategy of choice [4]. According to previous studies, the best time for TFTC is during the first year of life. For instance, Starr et al. recommended 6 months of age [5] and several other studies suggested the age of 3 to 11 months for the TFTC surgery [6, 7, 6, 8]. In contrast, in developing countries, the patients might remain undiagnosed until adulthood [9]. The main reasons for the late diagnosis of the disease are the late referral of patients due to the lack of complete knowledge (time of referral, whether the disease can be treated) about the disease. Furthermore, patients who do not have proper cardiac anatomy, suffer from severe cyanosis, have co-morbidities, undergo shunt implantation instead and complete surgery is delayed until they become older [4].

Addressing TOF at the adult age is challenging not only for the complications of undiagnosed TOF during the years (e.g., Myocardial dysfunction, ventricular arrhythmias, or even neurological complications) [9] but also for the possible difficulties during the surgery and the related issues [10]. In these patients, there is a possibility of multiorgan damage due to prolonged exposure to hypoxia [11–16]. In addition, due to the progressive hypertrophy of the right ventricle, there is a possibility of damage to the myocardium and a higher chance of arrhythmia [17]. Although the benefits of early and timely surgery are well known there is a possibility that these patients will be referred at an older age, especially in developing countries. If surgery is not performed for those who refer at an older age, their survival would be as high as 24% according to a ten-year study [4].

For this reason, current guidelines recommend total correction of the disease, regardless of the patient’s age [17]. Hence, the present study aimed to study the early outcome of TFTC, as well as possible complications in patients with tetralogy of Fallot who underwent surgery (TFTC) at the age of 15 and older in an Iranian tertiary heart center during a ten-year period (2010–2020).

## Methods

### Design

This study is a descriptive and retrospective study that was conducted on patients with tetralogy of Fallot aged over 15 years who underwent surgery. All data were taken from medical records at Shahid Rajaei Heart and Vascular Center (referral center for all types of heart diseases in Iran) between the years 2010 and 2020. Surgery including primary correction or second surgery after shunt palliation. All these patients were treated in the same center by the same surgical team, and their pre-and post-surgery evaluations were done by the adult congenital cardiologist. Before surgery, all patients underwent full evaluation (based on their condition) with CT-angiography; cardiac catheterization, or echocardiography (with the Vivid 7 device).

### Surgical technique

After median sternotomy and heparin injection, the aorta, superior vena cava (SVC), and inferior vena cava (IVC) were cannulated, and cardiopulmonary bypass (CPB) was started. Hypothermia was induced usually to 30 ºC but, in cases with high blood return from the left atrium (due to collaterals), deeper hypothermia was induced. The Modified Blalock Taussig shunt was closed (if present), and cardiac arrest was induced utilizing del Nido cardioplegic solution injection in the aortic root, repeated every 60 min. Then, VSD closure was done through the right atrial approach by a large piece of polytetrafluoroethylene (PTFE) patch utilizing interrupted pledgeted proline sutures. RVOT shaving was done usually via a longitudinal incision on the RVOT but in some cases was done through the tricuspid valve (if possible). The Main pulmonary artery (PA) was opened longitudinally and pulmonary valve commissurotomy was done and the size of the pulmonary valve annulus was measured (Z score ~ -2 considered acceptable). If the annulus was small, pulmonary valve replacement using a biologic prosthetic valve was done and the RVOT and main PA were augmented with an autologous pericardial patch (PA branch augmentation with a pericardial patch was done in the case of stenosis). The rest of the operation was done as routine, and the patient was separated from the cardiopulmonary bypass after rewarming.

If there was any doubt about the presence of residual pulmonary stenosis, the right ventricular pressure was measured by the transducer (the ratio of the right to the left ventricular pressure less than 0.7 is considered acceptable).

The patient’s medical records were examined and all necessary data were collected and recorded in the relevant checklist.

### Inclusion criteria

All patients diagnosed with tetralogy of Fallot who were operated on at the age of 15 years or older in our center were included in the study.

### Exclusion criteria

Patients whose medical records were incomplete were excluded from the study.

### Instrument

The data were collected from the patient’s medical records and entered into the checklist in four groups:

1. Demographical information including age at surgery, sex, use of RVOT patch during surgery, different types of cardiac arrhythmiaincluding atrial fibrillation (AF), Ventricular Tachycardia (VT), and frequent Premature ventricular contractions (PVCs) before surgery, associated anomaly such as Patent Ductus Arteriosus (PDA), Transposition of Great Arteries (TGA), Atrial Septal Defect (ASD), coronary anomaly and Major aortopulmonary collateral arteries (MAPCAs) greater than 3 mm [18] were detected and recorded, right and left ventricle ejection fraction, the severity of tricuspid and aortic regurgitation, pulmonary branch size (was defined as a small size of Mac Goon`s ratio < 1.2) in echocardiography, the severity of pulmonary artery and pulmonary branch stenosis(RVOT obstruction).
2. Early outcomes including in-hospital mortality, length of stay in the intensive care unit (ICU), bleeding after the surgery, and respiratory complications after the surgery such as pulmonary edema, pneumonia, etc.
3. One-year outcome including left ventricle ejection fraction (LVEF), right ventricle (RV) ejection fraction, the severity of tricuspid regurgitation (TR), and aortic regurgitation (AI) after surgery.

### Data analysis

For statistical analysis, we used Statistical Package for the Social Sciences (SPSS) software, version 22. We applied descriptive analysis for the description of the data.

### Ethical statement

Considering that the patients whose information was used in the study were not available, the code of ethics was received from the ethics committee of the hospital (IR.RHC.REC.1400.003).

## Results

In this study, according to the mentioned inclusion and exclusion criteria, 94 adult patients who underwent TFTC from 2010 to 2020 in Rajaie Cardiovascular Medical and Research Center were enrolled. The mean ± SD age of patients at surgery time was 26.7 ± 9.6 years. Additionally, 56 patients were male (59.6%), and 38 patients were female (40.4%), and the average age of male patients was significantly higher than females (P-value:0.009). demographic data and cardiac associated anomalies and problems (before surgery) were described in Table 1.

**Table 1:** Demographic data

*Variables of Demographic data*	*Frequency (Percent)*	*P-value*
**Age**		
***Mean***	26.7 ± 9.6 years	
***Age groups***		0.938
15–25 26–35 36–45 46–55	52 (55.3%) 27 (28.7%) 13 (13.8%) 2 (2.1%)	
**Gender**		**0.009**
Male Female	56 (59.6%) 38 (40.4%)	
**Cardiac anomalies**		0.024
Yes No	26 (27.7%) 68 (72.3%)	
**Sidedness**		0.216
Right Left	24 (25.5%) 70 (74.5%)	
**Arrhythmia**		0.205
Yes No	17 (18.1%) 77 (81.9%)	
**MAPCA (Major aortopulmonary collateral arteries)**		0.203
Yes No	38 (40.4%) 56 (59.6%)	
**RVOT patch**	91 (96.8%)	

**Table 2:** Outcomes

Variable	Frequency (Percent)
**In-hospital mortality**	5 (5.3%)
**ICU admission**	
**Mean** More than 48 h Less than 48 h	87 ± 30 h 92 (97.9%) 2 (2.1%)
**Respiratory complications**	14 (14.9%)
**Bleeding after surgery**	20 (21.5%)

The mean ± SD total perfusion time and aortic cross-clamp time were 126.44 ± 29.75 and 95.22 ± 21.38 min, respectively. In addition, in 91 (96.8%) patients, the surgeon used an RVOT patch during correction.

We investigated the early outcomes, including in-hospital mortality, ICU length of stay, bleeding after surgery, and respiratory complications. In-hospital mortality was 5.3%. Two patients died due to sepsis and multi-organ failure after the operation (both had pre-operative pulmonary valve endocarditis). Three patients also died due to severe right ventricular failure followed by multi-organ failure despite ECMO implantation. The mean ± SD ICU length of stay was 87 ± 30 h. The ICU length of stay was more than 48 h in 92 (97.9%) patients, and only 2 (2.1%) patients were transferred to the ward earlier than this time. Post-operative bleeding was reported in 20 patients (21.5%), of which 18 (90%) needed surgical intervention. The cause of bleeding was surgical in 3/18 (16.66%), including bleeding from the RVOT patch in 2 patients and from the SVC cannulation site in 1 patient, and non-surgical in 15/18 (83.33%) patients (diffuse bleeding due to coagulopathy and presence of multiple small collaterals in the surgical dissection sites). Multiorgan failure was seen in five patients unfortunately all died (Two of them had preoperative pulmonary valve endocarditis, and the other three had postoperative RV failure). Two patients also had postoperative acute kidney injury that was successfully managed. No neurological complications were observed.

One-year outcomes included the right and left ventricular dysfunction, AI, TR, and RVOT obstruction severity. These data were compared in the patients before and after the surgery. The prevalence of TR significantly decreased after the surgery (P-value: 0.006). Pulmonary branch size before and after the surgery were hypoplastic or small in 17 (18.1%) and 3 patients (3.2%), respectively and it was significantly acceptable after the surgery (P-value: 0.000). In the subgroup analysis for comparison of the small and hypoplastic pulmonary branches with the normal situation, no significant difference was found according to the patient’s primary and secondary outcomes (Table 3].

**Table 3:** The results of the surgery

Variable	Before surgery	After surgery	P-value
**RV dysfunction**			0.338
Normal Mild Moderate Severe	6 (6.4%) 39 (41.5%) 44 (46.8%) 5 (5.3%)	1 (1.1%) 32 (34%) 54 (57.4%) 7 (7.4%)	
**LV dysfunction**			0.062
LVEF > 50% LVEF: 35–50% LVEF < 35%	52 (55.3%) 38 (40.4%) 4 (4.3%)	37 (39.4%) 52 (55.3%) 5 (5.3%)	
**Tricuspid Regurgitation (TR)**			**0.006**
Mild Moderate Severe	69 (73.4%) 23 (24.5%) 2 (2.1%)	53 (56.4%) 36 (38.3%) 5 (5.3%)	
**Aortic Regurgitation (AR)**			
Normal Mild Moderate Severe	30 (31.9%) 62 (66%) 2 (2.1%)	18 (19.1%) 69 (73.4%) 6 (6.4%) 1 (1.1%)	
**Pulmonary size**			**0.000**
Acceptable Hypoplastic or small	77 (81.9%) 17 (18.1%)	91 (96.8%) 3 (3.2%)	

Before surgery, mild, moderate, and severe RVOT obstruction were found in 2.1%, 3.1%, and 94.6% of the patients, respectively. After the surgery, RVOT obstruction was resolved in 50.5% of the patients. Also, mild and moderate RVOT obstruction was found in 39.3% and 12.7% of the patients, respectively. Changes in RV and LV function before and after surgery was described in Table 3.

## Discussion

In this study, we intended to investigate short complications as well as the improvement of cardiac function factors in adult patients (older than 15 years) with Tetralogy of Fallot who underwent surgery. The mean ± SD age of the study group at the time of surgery was 26.7 ± 9.6 years. Similarly, Atik et al. reported patients age of 26.6 ± 11.1 years at the time of TFTC [11], while, Presbitero et al. and Charles et al. reported higher mean age at the time of TFTC [14, 19] and Erdoğan et al. and Imran Khan et al. described younger patients who underwent the surgery [4, 20]. This age difference in different studies can be due to the difference in diagnostic methods and the lack of awareness among patients for timely referral, which itself requires special attention.

In this study the prevalence of male patients was higher, the same as in most other previous studies [9, 11, 14, 20]. Only in one study, the prevalence of female patients were higher [11]. Right-sidedness was found in 25.5% of our patients, but in the other studies, it was higher than in our study [4, 19]. Also, cardiac anomalies in our patients were higher than Chlarles et al. study [19]. In general, it is recommended that all patients with tetralogy of Fallot, especially at an older age, undergo a complete examination for associated anomalies before surgery, because it can have a significant impact on the type of surgery and the course of treatment.

Arrhythmias were diagnosed in 18.1% of the patients. In two studies, the prevalence of arrhythmias was higher than in our study [11, 14]. In the study of Imran et al., arterial arrhythmias and heart block were found in 10% of the patients [4]. In addition, in the study of Charles et al., bundle branch block (BBB) was found in more than half of the patients [19]. MAPCAs were diagnosed in 40.4% of the patients and it was higher than Khan et al. and Khalid et al. studies [4, 9]. One of the reasons for the higher prevalence of MAPCAs in our study could be the older age of our study patients compared to other studies, which increases the size of MAPCAs that can be identified in CT angiography.

In the first study on adult patients with TOF in 1972 by Charles et al. the mortality rate was high. Four deaths were reported out of seventeen surgeries (23%) [19] that may be occurred due to the limited number of patients and the surgical technique issues at that time. During recent decades, the mortality rate of TFTC surgery in adult patients has decreased significantly [21]. In most of these studies, the mortality rate ranged from 1.8 to 8% [4, 9, 11, 14, 20, 22]. In our study, in-hospital mortality was 5.3% but in children who underwent TFTC at the recommended time, the mortality rate is lower than 2% [5]. One of the reasons for higher mortality in patients who underwent surgery at an older age can be the presence of more severe right ventricular failure. It is because the right ventricle had been exposed to pressure overload for a longer period, and even removing the right ventricular outflow tract stenosis cannot resolve ventricular remodeling. Also, the number of muscles that are cut and removed during RVOT shaving is more in these cases because of the severe RV hypertrophy that can lead to more degrees of RV dysfunction. The presence of ventricular failure will increase the risk of arrhythmia and the symptoms of systemic congestion and, as a result, increase mortality. In most of the recent studies, the mortality rate was lower than in our study, and just in one study, the mortality rate was higher [4]. In general, it can be said that based on the studies [23], if patients with tetralogy of Fallot are operated on time, the risk of mortality is lower and the long-term survival is excellent, compared to patients who are operated on at an older age. The most important issue in this field is the complete elimination of all defects and timely preservation of the myocardium, as well as the correct and timely treatment of surgical complications. According to these cases, it can be said that one of the reasons for the higher risk of mortality and surgical complications at the older age is the prolonged time of anatomical, hemodynamic, and physiological cardiac disturbances (caused by the heart defects of the disease itself), which affects the right and left ventricular function. In addition, with the increasing age of patients, the probability of collateral formation as well as the occurrence of accompanying arrhythmias and coagulopathies caused by hypoxia rises, which increases the chance of post-surgery complications and mortality compared to surgery at a younger age. However, in our study, none of these factors had a significant effect in terms of statistics, which could be due to the small sample size.

In our study, the mean ± SD ICU length of stay was 87 ± 30 h. The result of the other studies was approximately similar to our study [9, 20], but Atik et al. reported shorter ICU stay time [11].

In our study, postoperative bleeding was reported in 21.5% of our patients and most of them needed a surgical approach for bleeding control. In the study of Khalid et al. 2.9% of the patients needed another surgery due to tamponade or bleeding and residual VSD was reported in 8.7% of the cases, but none of them were moderate or severe [9]. Bleeding was the reason for 3.8% of reoperations in the patients in the Khan et al. study [4].

Respiratory complications were found in 14.9% of the patients. In the study of Erdoğan et al., 2.4% of the patients needed mechanical ventilation for more than three days [20]. Atik et al. reported that 7.7% of the patients needed long mechanical respiratory support [11]. As Khan et al. reported in their study, postoperative complications encountered were low cardiac output syndrome (11.25%), pleural effusion requiring tapping (3.75%), reoperation for bleeding (3.8%), pulmonary regurgitation (moderate to severe) (25%) which occurred in the transannular patch group only and atrial arrhythmia (5%). In addition, most of the results of the surgery including the correction of TR, pulmonary size, RVOT obstruction, and pulmonary branch size were successful.

This study showed that although the patients with TOF were diagnosed and underwent surgery too late in comparison with the guidelines, the results were acceptable. As mentioned above, TR was treated significantly. Also, the results of the surgery for improving the pulmonary stenosis, treatment of RVOT obstruction, and pulmonary branch size were excellent. Before surgery, severe RVOT obstruction was found in most of the patients. After surgery, it was resolved completely in about half of the patients. Also, severe obstruction was not found in any of the patients after the surgery. The results of the previous studies for TFTC in the patients who were older than the recommended age, was successful [24, 25].

As mentioned in the previous parts, the gold standard time for the management of the patients with TOF is during the first year of life. Most of the patients with TOF in developed countries are diagnosed and managed in childhood, so there is a lack of data about the newly diagnosed adult patients in these countries. In addition, studies about the management of TOF in adulthood in developing countries are limited, so this study can help us for choosing a better approach to this special group of patients. Our study confirmed that TFTC surgery in adulthood is safe with acceptable outcomes and limited complications.

In a study conducted by Ignacio Lugones et al. [26], it was shown that the short-term results of surgery of TOF in adult patients depend on the cardiac anatomy and the clinical conditions of the patient before surgery, so it is recommended in adult patients, any types of the valvular heart diseases and cardiac defect should be corrected during the TFTC surgery. Despite the young age of our study population, the age-related risk of coronary artery disease will play an important role in the long-term outcome of the patients. Positive history of TFTC in an adult patient should not put a shadow on the possibility of age-related other cardiovascular disorders. In other words, we must consider routine cardiovascular screenings in this patient population.

## Conclusion

Although it is better to perform TFTC in childhood, age is not a contraindication for surgical management. Moreover, according to the high rate of late diagnosis of TOF in developing countries, TOF should be kept in mind as a potential differential diagnosis in children and adults who have suspicious symptoms and signs. In addition, knowing that accompanying anomalies can affect the course of the disease and the type of surgery, careful preoperative assessment is recommended.

## Data Availability

The data that support the findings of this study are available from the corresponding author upon reasonable request.

## References

[1] Moazenzadeh M, Jafari F, Farrokhnia M, Aliramezany M (2020). First reported case of unrepaired tetralogy of Fallot complicated with coronavirus disease-19 (COVID-19). Cardiol Young.

[2] Bailliard F, Anderson RH (2009). Tetralogy of fallot. Orphanet J Rare Dis.

[3] Anderson RH, Sarwark A, Spicer DE, Backer CL (2014). Exercises in anatomy: tetralogy of Fallot. Multimedia Man Cardiothorac Surgery: MMCTS.

[4] Khan I, Tufail Z, Afridi S, Iqbal M, Khan T, Waheed A (2016). Surgery for tetralogy of Fallot in adults: early outcomes. Brazilian J Cardiovasc Surg.

[5] Starr JP (2010). Tetralogy of Fallot: yesterday and today. World J Surg.

[6] Van Arsdell GS, Maharaj GS, Tom J, Rao VK, Coles JG, Freedom RM (2000). What is the optimal age for repair of tetralogy of Fallot?. Circulation.

[7] Vickers NJ (2017). Animal communication: when i’m calling you, will you answer too?. Curr Biol.

[8] Steiner MB, Tang X, Gossett JM, Malik S, Prodhan P (2014). Timing of complete repair of non–ductal-dependent tetralogy of Fallot and short-term postoperative outcomes, a multicenter analysis. J Thorac Cardiovasc Surg.

[9] Khalid ZR, Mughal AR, Siddiqui MMA, ul Haq R (2020). Early outcome of total correction in adult tetralogy of fallot patients. Prof Med J.

[10] Kirklin J. Ventricular septal defect and pulmonary stenosis or atresia. Cardiac surgery. 1993:863 – 85.

[11] Atik FA, Atik E, da Cunha CR, Caneo LF, Assad RS, Jatene MB (2004). Long-term results of correction of tetralogy of Fallot in adulthood. Eur J Cardiothorac Surg.

[12] Nollert G, Fischlein T, Böhmer C, Dewald O, Kreuzer E, Welz A (1997). Long-term results of total repair of tetralogy of Fallot in adulthood: 35 years follow-up in 104 patients corrected at the age of 18 or older. Thorac Cardiovasc Surg.

[13] John S, Kejriwal N, Ravikumar E, Bashi V, Mohanty B, Sukumar I (1986). The clinical profile and surgical treatment of tetralogy of Fallot in the adult: results of repair in 200 patients. Ann Thorac Surg.

[14] Presbitero P, Demarie D, Aruta E, Villani M, Disumma M, Ottino G (1988). Results of total correction of tetralogy of Fallot performed in adults. Ann Thorac Surg.

[15] Dittrich S, Vogel M, Dähnert I, Berger F, Lange PR, Alexi-Meskishvili V (1999). Surgical repair of tetralogy of Fallot in adults today. Clin Cardiol.

[16] Deanfield J, McKenna W, Presbitero P, England D, Graham G, Hallidie-Smith K (1984). Ventricular arrhythmia in unrepaired and repaired tetralogy of Fallot. Relation to age, timing of repair, and haemodynamic status. Heart.

[17] Ramanan S, Sasikumar N, Manohar K, Ramani SS, Kumar RS, Agarwal R (2019). Adult tetralogy repair: factors affecting early outcome in the current era. Asian Cardiovasc Thorac Annals.

[18] Alex A, Ayyappan A, Valakkada J, Kramadhari H, Sasikumar D, Menon S (2022). Major aortopulmonary collateral arteries.

[19] Higgins CB, Mulder DG (1972). Tetralogy of Fallot in the adult. Am J Cardiol.

[20] Jayasankar V, Woo YJ, Pirolli TJ, Bish LT, Berry MF, Burdick J (2005). Induction of angiogenesis and inhibition of apoptosis by hepatocyte growth factor effectively treats postischemic Heart Failure. J Card Surg.

[21] Llamosas-Falcón L, Bermejo-Sánchez E, Sánchez-Díaz G, Villaverde-Hueso A, Posada de la Paz M, Alonso-Ferreira V (2019). Tetralogy of Fallot in Spain: a nationwide registry-based mortality study across 36 years. Orphanet J Rare Dis.

[22] BEACH GA JR, MALM JR. Total correction of tetralogy of Fallot in adolescents and adults. Circulation. 1971;43(5s1):I-37-I-43. 4102928

[23] Hashemzadeh K, Hashemzadeh S. Early and late results of total correction of tetralogy of Fallot. Acta Medica Iranica. 2010:117–22. 21133005

[24] Heinisch PP, Guarino L, Hutter DM, Bartkevics M, Erdös G, Eberle B (2019). Late correction of tetralogy of Fallot in children. Swiss Med Wkly.

[25] Mottier V, Prša M. Contemporary early results of late repair of tetralogy of Fallot in children: a single-centre case series. Swiss Med Wkly. 2021(13). 10.4414/smw.2021.20491 33934315

[26] Lugones I, Inguanzo PD, Antoni D, Di Santo M, García RO. Surgical correction of tetralogy of fallot in adulthood. 2012.

